# Swimming Stroke Mechanical Efficiency and Physiological Responses of 100-m Backstroke with and without the use of paddles

**DOI:** 10.2478/hukin-2014-0019

**Published:** 2014-04-09

**Authors:** Spilios Messinis, Nikos Beidaris, Spyros Messinis, Helen Soultanakis, Petros Botonis, Theodoros Platanou

**Affiliations:** 1 School of Physical Education and Sport Sciences, Department of Aquatic Sports, University of Athens, Greece

**Keywords:** swimming, training aids, stroke length, stroke number, gliding length, blood lactate

## Abstract

The use of swimming aids during training contributes to greater swimming efficiency by the improvement of the swimming specific power of the athlete. The purpose of this study was to compare the swimming stroke technical characteristics and the physiological responses of swimming 100-m backstroke, with and without the use of paddles at maximum and sub-maximum intensities at the same swimming speed. Eight swimmers competing at the national level participated in this study. The measurements took place at 4 different sessions. At every session, each participant swam individually one 100-m backstroke swimming trial with or without paddles at the same speed and two levels of intensity (100% and 85% of maximum speed). The results revealed lower stroke length, greater stroke number and gliding length without the use of swimming paddles at both intensities. Blood lactate concentration (10.03±2.96 vs. 5.85±2.23 mmol/l) and Rating of Perceived Exertion (17.43±2.07 vs. 12±2.82) were greater without the use of swimming paddles only at 100% of maximum speed. Thus, swimming backstroke with paddles compared to unaided swimming, at a similar speed, showed a greater efficiency at maximal but not at sub-maximal intensity.

## Introduction

In contemporary sports science the development of training and kinetic models, thorough knowledge of biomechanics and of the forces developed upon and from the athlete, have contributed to the advancement of the training process and the improvement of all parameters related to performance. The use of swimming aids during training contributes to greater swimming efficiency by improving specific power of the athlete. In the present study, the mechanical efficiency and energetics of backstroke swimming with and without the use of paddles, were evaluated. Swimming paddles are plastic plates applied with “ties” around the palm of the swimmer’s hands, to ensure a tight and solid contact. They come in several shapes and sizes and the larger the size the higher the level of difficulty. The swimming paddles are recommended to swimmers, for the development of their power within water to specific stroke patterns used. In swimming with paddles, the trainee is required to overcome the resistance created by the water due to a greater surface area. As a consequence, by using a greater surface area a better performance is achieved by the athlete, i.e. his speed is increased at a predetermined distance (Gourgoulis et al., 2006; Toussaint et al., 1991), thereby achieving greater propulsive distance (Ogita and Tabata, 1993).

The coaches during training usually set the intensity of swimming with paddles by using as an index the same intensity of time performance (average speed) as that swimming without paddles. Several comparative studies have been carried out in freestyle swimming, for the determination of energy demands and the biomechanical parameters of swimming with and without swimming paddles, with the athletes usually swimming a fixed number of strokes at a predetermined distance (Ogita and Tabata, 1993; Ogita et al., 1999; Toussaint et al., 1991). However, little is known about the technical characteristics and physiological responses in backstroke swimming with paddles in comparison to that without swimming paddles, when keeping the swimming speed constant in both cases.

The physiological demands and performance characteristics of an effort are important when the coach needs to analyse the training data and gain feedback for the usefulness of any training method and training goal achievement. It is widely known that heart rate, blood lactate concentration and maximum oxygen uptake are determinants of exercise intensity (Billat et al., 1994; Billat, 1996; Chen et al., 2002; Colwin, 2002; Hofmann et al., 1994; Holmer et al., 1974; Howley et al., 1995; Fernandez et al., 2003; Niewiadomski et al., 2007; Obert et al., 1992; Rivera-Brown et al., 2000; Smith et al., 2002; Ueda and Kurokawa, 1991; Weltman et al., 1990).

The determination of exercise intensity with paddles by taking as a measure the determination of time performance within a given distance without swimming paddles may have a different effect on physiological responses due to the possible greater power applied by paddles and the higher speed which the swimmers may develop. On this basis, the present study is designed to examine the physiological responses and the characteristics of technique in backstroke swimming with and without the use of swimming paddles at a given speed. This study will make reference to two different swimming intensities, at maximum and sub-maximum speed. These comparisons will help in the evaluation and extraction of useful conclusions for the design of training programs and will identify the role of swimming paddles, as a means of improving the efficiency of the athlete in the training process. Specifically, it is possible to hypothesize that differences will be elicited in the mechanical swimming efficiency of backstroke by swimming either with or without paddles at a similar speed and at two different intensities.

The purpose of this study was to compare the swimming stroke technical characteristics and the physiological responses of swimming 100-m backstroke, with and without the use of paddles at maximum and sub-maximum intensities at the same swimming speed.

## Material and Methods

### Participants

Eight well trained swimmers competing at the national level participated in this study. Backstroke was their principle or secondary competitive swimming style. Data were collected in a 25-m indoor swimming pool. The temperature of the water was 26 ± 0.1°C, the ambient temperature was 28 ± 1°C and the relative humidity was 55% in all testing and training conditions. Measurements took place during the morning hours after collection of participants’ anthropometric characteristics. Participants’ anthropometric characteristics are depicted in Table 1. Each subject, prior to measurements, was informed of all possible risks associated with the study and signed an informed consent form. The employed design was approved by the Institutional Review Board for use of Human Subjects, in accordance with the ethical standards and the Declaration of Helsinki.

### Design

This study took place at 4 different sessions, each separated by 48 hours, one month prior to their formal competitive season. At every session after an initial warm-up (800-m), every participant swam individually a 100-m backstroke swimming trial with or without paddles at the same speed at maximum and sub-maximum intensity (100% and 85%). Each condition was performed on a different day in a random cross-over design. Participants were instructed to arrive at site without prior consumption of food or drink.

During the 1st day they swam 100-m backstroke at maximum speed (100%) without paddles. After two days they repeated the same trial with medium size paddles (Fahnemann swimpower 19×16 cm, Fahnemann Germany), at the same speed as the trial without paddles. Speed was established by the use of a novel piece of equipment that was designed and validated for this study specifically for backstroke and a visual cue (Messinis and Platanou, 2010). After determining speed (m/sec) in the first maximum backstroke swim without paddles, swimmers were able to replicate the same rate with the paddles by following a marked point that was moving on a cable suspended along the middle of the swimming lane. This point was electronically programmed to “run” on the required speed of the time that was established on the initial 100m trial and by following the marked point the swimmer was able to swim at a selected speed. On the third day the sub-maximal (85% of maximal speed) intensity of the 100-m backstroke trial was determined through the use of the preceding maximal performance test. Then swimmers swam a third 100-m backstroke trial without swimming paddles at a sub-maximal speed and on a final and fourth day they swam a 100-m sub-maximal test with swimming paddles at the same speed as that pre-established without paddles. For all 4 trials, participants were continuously monitored for heart rate and for stroke characteristics as well as time during swimming, for oxygen uptake (VO_2_) and lactate concentration before and after the trials. Additionally, ratings of swimmers’ perceived exertion (RPE) were evaluated after all trials.

### Heart Rate, VO_2max_, RPE and Blood Lactate Determinations

Heart rate of every participant was recorded every 5 seconds during swimming trials with a continuous Polar monitor system (Polar 610i, Finland) with a Polar belt secured around their chest and a receiver secured on their bathing suit. In the end of every trial expired air was collected for 2 minutes at recovery, to determine oxygen uptake (VO_2_) via a validated automatic gas analyser (VO 2000 Breeze Lite, Medical Graphics Corp., USA). The first breath immediately after swimming was collected by having the swimmer exhale in the mouthpiece connected to the automatic analyser while applying a nose clip. At the end of the trial the swimmers were also asked to show their perceived exertion on a 6–20 Borg scale (Borg, 1982). For the measurement of blood lactate concentration, fingertip blood samples were collected at the 3rd, 5th and 7th minute of recovery and immediately analysed by the automated lactate analyzer (Accusport, Boehringer, Germany).

### Stroke and Performance Characteristics

A SVHS video camera (Panasonic MS5, Japan) (50Hz, 720X576 resolution) located perpendicular to the swimmer’s motion was used to record all swimming trials for time performance and stroke characteristics. Thereafter the recorded data were analysed for the determination of the stroke rate, stroke length and gliding length for every 100-m backstroke. All strokes required to cover the 100-m distance for all trials were counted for stroke number determination. The “gliding length” was the distance the swimmer covered from the wall of the pool, up until the initiation of the first arm stroke. Mean stroke length was calculated by subtracting the “gliding” distances from the total distance covered and by then dividing it by the total strokes that were required to cover the distance.

### Constant rate determination

**Figure 1.**
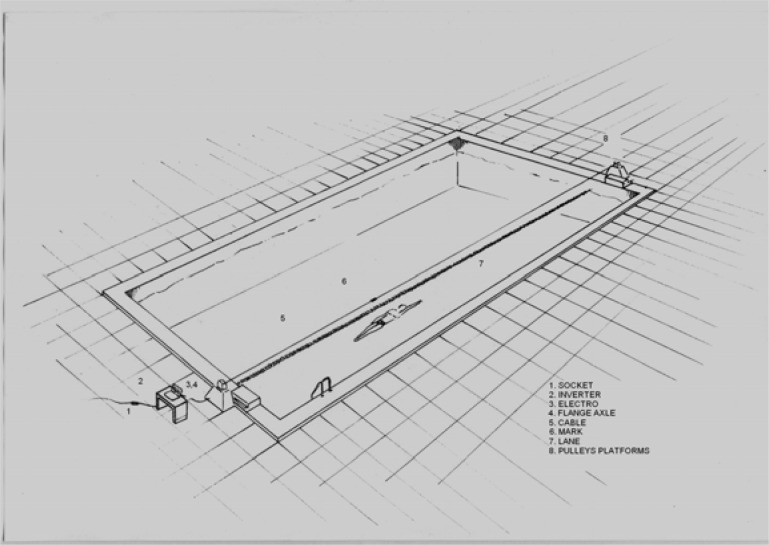
The experimental setting with the apparatus used to control the swimming speed.

The maintenance of a constant rate between trials was established through a device that was constructed and validated for the specific needs of this study (Messinis and Platanou, 2010). The construction of this device was based on one previously built by Clinton (1964). A base of electrically driven pulleys was placed at the edge of one side of the pool and connected to another base on the other side of the 25-m pool, suspending a rotating cable 150-cm above water (Figure 1). A visible mark was attached on the cable and would lead the backstroke swimmers to swim at a pre-selected speed by following the marker. An exponential regression equation was used for the adjustment of the motor revolutions with the corresponding speed. The evaluation of validity and reliability of the motor construction was tested on repeated measurements. The time taken for the coloured mark to complete each 50-m distance was recorded for 50 consecutive repetitions under five randomly selected numbers of revolutions (Messinis and Platanou, 2010). The time of each complete 50-m cycle was recorder using a digital chronograph (Casio, Japan).

### Statistical analysis

Results are presented as mean ± standard deviation (SD). A two-way analysis of variance (ANOVA) for repeated measures was used for comparison of physiological and mechanical parameters between the experimental conditions (2 conditions × 2 speed steps). Significant differences between means were located using the Tukey post-hoc test. The evaluation of validity and reliability of the motor construction was tested on repeated measurements and the coefficient of variation was determined. Additionally, the time taken of the rotating coloured mark was compared with the time taken of a group of swimmers to complete 100-m using the t-test for independent samples. The coefficient of variation of the swimming performance time and time of the rotating mark was calculated. The accepted level of significance was set at p<0.05.

## Results

### Reliability and validity of the mechanical construction

The construction showed a high degree of reliability (r=0.99, p<0.05). The exponential relationship and the prediction equation y=82542x^−1,015^ where x (performance time) and y (revolutions) describe the relationship between time and number of revolutions. This equation was used for the evaluation and calibration of the mechanical construction. The time recorded after a 50 m rotation of the colored mark at 1200, 1600, 1800, 2000 and 2200 revolutions was 64.66±0.56, 48.50±0.22, 43.21±0.42, 39.07±0.37 and 35.60±0.19 s with a coefficient of variation (CV) of 0.31%, 0.05%, 0.18%, 0.14% and 0.04%, respectively. The low CV indicates the homogeneity of the data collected and low speed variability of the rotating mark. The CV of the swimmers performance time on repeated 50 m test was 0.16% and no difference was observed between the time of the rotating colored mark and the swimmers time in the 100 m distance (89.82±14.28 and 89.66±14.29 s, respectively, p>0.05).

### Performance Characteristics

Performance and stroke characteristics are presented in Table 2. The performance characteristics reveal differences between swimming with and without paddles at both intensities, except in the gliding length at the 85% level of intensity. Specifically in swimming without paddles the number of strokes and the gliding length were significantly greater, while the mean stroke length was significantly lower when compared to swimming with paddles.

### Physiological Responses

**Figure 2.**
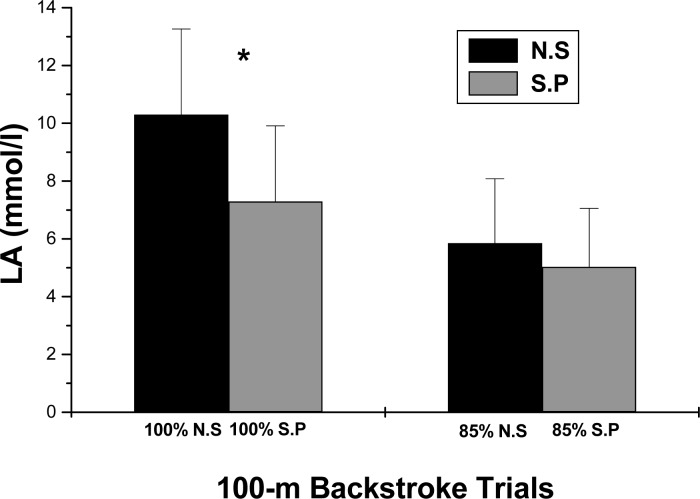
Peak lactate concentration values (means+SD; mmol/l), of 100-m backstroke swimming with (S.P) and without (N.S) paddles, at 100% and 85% of maximum speed. Asterisks (^*^) denote differences between 100 %-speed trials.

**Figure 3.**
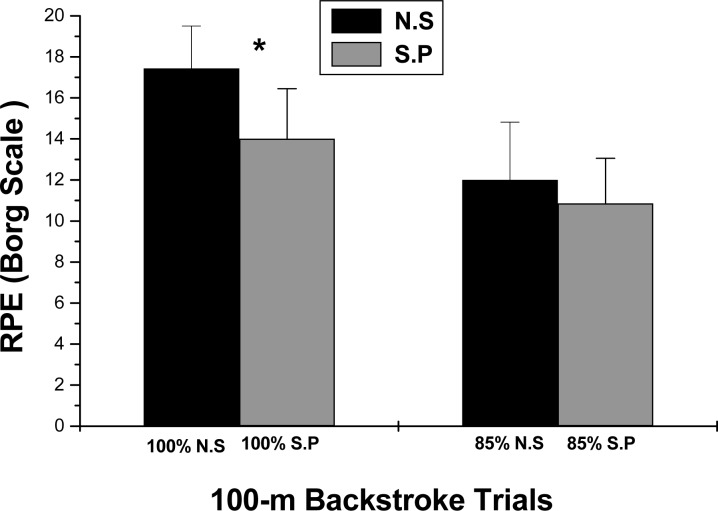
Ratings of Perceived Exertion (means+SD; 6–20 Borg scale), of 100-m backstroke swimming with (S.P) and without (N.S) paddles, at 100% and 85% of maximum speed. Asterisks (^*^) denote differences between 100 %-speed trials.

Peak blood lactate levels were significantly greater only after the 100% maximum velocity trial of swimming with no paddles. No differences in post exercise peak lactate concentrations were observed between the 85% velocity trials with (S.P.) or without (N.S) paddles (Figure 2). Rating of perceived exertion was also only greater after the maximum 100%-velocity trial without the use of paddles, but with no differences at the lower intensity (Figure 3). VO_2_ at 100%-velocity (N.S.: 51.07±16.26 vs. S.P.: 49.14±22.82 ml·kg^−1^·min^−1^), and at 85%-velocity (N.S.: 33.11±6.30 vs. S.P.: 30.82±7.25 ml·kg^−1^·min^−1^) exhibited higher trends without the use of paddles, but these differences were not significant. Heart rate at both intensities, i.e. at 100% (N.S: 179±9.14 vs. S.P.: 175.8±10.43 beats/min) and at 85% (N.S.: 163.4±15.57 vs. S.P.: 156.4±19.71 beats/min), also showed similar higher trends without the use of paddles which were not significant.

## Discussion

In the present study the physiological responses and certain biomechanical characteristics of backstroke swimming with and without paddles at two different intensities, at maximal (100% velocity with no paddles) and submaximal velocity (85% of maximal velocity with no paddles) were investigated. The results showed that at maximal effort the energy cost is smaller when swimming backstroke with paddles as evident by the smaller lactate production, lower HR and lower oxygen uptake at recovery. On the other hand at sumbaximal efforts (85% of maximal velocity), certain stroke characteristics changed but no physiological differences were observed.

### Biomechanical characteristics

In the technical features that were evaluated, one of the main findings was that the number of strokes with paddles was smaller than without paddles at both intensities. In agreement to Payton and Lauder (1995) the stroke frequency is affected from the increase of the surface area that contacts water with the use of certain swimming aids. In swimming with paddles, swimmers can pull more water due to the greater pulling surface area that the paddles provide (Gourgoulis et al., 2006; Ogita and Tabata, 1993). The same views are shared by Maglisho (2003) and Toussaint et al. (1991) who add that the increase of the propulsive surface area contributes to the increase of swimming speed and concomitantly decreases the stroke frequency. It is interesting to note that in the present study the smaller number of strokes covered a larger distance if one takes into consideration that the gliding distance was smaller and the swimming distance was greater when using the paddles than without. The mean gliding length in swimming with paddles was 18.09±5.14 m versus 23.88±6.10 m without paddles, thus the total length swum after subtracting the gliding length was greater.

The smaller number of strokes with the paddles was compensated by the greater stroke length that was observed in this study as opposed to a shorter stroke length and greater number of strokes when the swimmers did not use the paddles. These results are in agreement with Toussaint’s et al. (1991) research that also showed that when swimming 100-m freestyle at a constant speed with paddles as opposed to without, the distance per stroke cycle was greater and the stroke frequency smaller (Toussaint et al., 1991). However there are other studies that have demonstrated either a decrease in stroke length (Payton and Lauder, 1995) or a maintenance of it (Zafiriadis et al., 2007).

### Physiological Responses

The physiological variables that were measured and evaluated were heart rate, VO_2_ and blood lactate concentration of swimmers during 100-m backstroke swimming with and without swimming paddles at two different intensities. Additionally, ratings of swimmers’ perceived exertion were evaluated after all trials. The findings of the present study showed certain significant differences of physiological responses only after maximal trials (100% effort), manifested as lower lactate levels with the use of paddles, and a lowering tendency of oxygen uptake and heart rate, but not after submaximal efforts. Participants also demonstrated higher values at the Borg scale, when swimming without paddles only after the maximal velocity (100%) trial. This was possibly due to the fact that the intensity of the swimmers’ efforts at 100% velocity was greater when one takes into consideration the greater production of blood lactate. Therefore, the work produced when swimming without paddles at 100% velocity appears to be less efficient than swimming with paddles and is possibly due to a greater number of less propulsive strokes required to cover the same distance.

However, at trials of 85% of maximal velocity, with and without paddles, RPE and all the indices of intensity (blood lactate concentration, HR, and VO_2_ at recovery) did not demonstrate any significant differences. Therefore, the slower speed did not affect the physiological responses when using a swimming aid in comparison to free swimming. It is widely accepted that lactate concentrations serve as indices of intensity (Billat, 1996; Howley et al., 1995). In the present study blood lactate concentrations at maximal intensity, were significantly higher in backstroke swimming without paddles than swimming with paddles. On the other hand at submaximal intensity there were no differences between the two different exercise conditions. Swimming at a lower intensity with or without paddles was not adequate to activate anaerobic lactate metabolism to the extent that possible differences would be elicited. Lactic acid is produced during exercise when the rate of glycolysis is increased and is always relative to the intensity and duration of exercise. Previous data have demonstrated that as speed increases lactate concentration also does (Maglischo, 2003; Smith et al., 2002). From the results of the present study we can conclude that since time greatly differs between maximal and submaximal trials, the rate of substrate mobilization also changes considerably. In other studies measuring blood lactate concentration with and without paddles during freestyle swimming, even though they found no differences, it is noteworthy to mention that while speed was maximum at both trials it was not similar, as in the present study (Ogita and Tabata, 1993; Sidney et al.,1996). In another comparative study in backstroke swimming seeking for lactate concentration differences between another type of aid (a pulling water parachute) and unaided swimming, by keeping the stroke rate similar, no differences were reported at two different intensities, which did not however reach maximal values (Zafiriadis et al., 2007).

Recovery VO_2_ which was measured in the present study evaluates the aerobic energy contribution during exercise. The O_2_ consumed after swimming during recovery and which is in excess of the required at a resting state is known as oxygen debt. It expresses the cardiorespiratory and muscular capacity of the system and its ability to consume O_2_ during a given time segment. The O_2_ uptake increases as the intensity of exercise increases in a linear manner up to a point, when it will plateau despite an increase in work output. In the present study although there appeared to be an increasing tendency of O_2_ uptake without the use of paddles at both intensities, it was not significant. This is contrary to a study by Ogita et al. (1999), who found that in swimming without paddles oxygen uptake was higher when compared to swimming with swimming paddles in freestyle. This is possible due to the limited amount of time covered in swimming 100-m backstroke, that was not adequate to intensely activate the aerobic pathways, at least to the extent of demonstrating great differences. Heart rates, even if they showed some higher trends at the greater intensity, those were not significant when swimming with and without paddles. The same observation was done by other researchers (Ogita and Tabata, 1993; Sidney et al., 1996).

In accordance to the above and taking into consideration our initial research hypothesis, we observe that even if heart rate and oxygen uptake demonstrated slight differences between conditions (with paddles and without paddles) at both intensities, those differences were not significant. The higher blood lactate concentration of the swimmers without paddles after the maximum performance confirms our initial hypothesis, while during submaximal efforts with and without paddles, there were no significant differences. At submaximal speeds the improvement of efficiency was only evident on biomechanical, stroking variables with no concomitant shifts in physiological responses. Swimming at 100% intensity with paddles was at the same speed as with no paddles. It has been shown that when swimmers use paddles they are able to further increase their speed compared to that achieved with no paddles (Gourgoulis et al., 2006). Therefore, the swimmers probably exercised at a lower relative intensity at both 85% and 100% conditions while using the paddles. However, the same percentage of the relative intensity drop of swimming speed, in the conditions with the paddles, has a much greater impact on the energy cost at the higher compared to the lower speed. This can be clearly explained by the curvilinear relationship of speed versus energy cost in swimming (Holmer, 1974). This may explain why no differences were observed in the 85% as those observed at the 100% intensity.

Our hypotheses were also confirmed as far as biomechanical kinematic characteristics of strokes were concerned, by observing differences in stroke length, stroke frequency and gliding length with and without the use of swimming paddles. Thus, the use of a swimming aid such as swimming paddles at a similar speed to unaided swimming, has an effect on both, the technical and mechanical parameters of backstroke as well as on certain physiological responses which overall contribute to greater efficiency when using the paddles particularly at maximum speeds.

The present study is designed only for backstroke swimming. It would have been of great value to investigate the physiological and biomechanical characteristics of the other swimming styles and competitive events at various distances and at similar speed with and without paddles. Limitations of this study included: a) participants were not elite swimmers but trained swimmers, b) this was only in application to backstroke and to the 100-m distance and, c) backstroke was not the preferred stroke of all participants.

## Conclusion

The novel findings of this study were that swimming backstroke with paddles compared to unaided swimming at a similar speed showed a greater efficiency at maximum but not at sub-maximal speed. Swimming with paddles probably reflects an underestimated intensity compared to no-paddles. In applying the above data to a swimming training design of sets with swimming paddles, coaches need to take into consideration the differences in energetics and not base their design on swimmers’ responses when swimming without paddles. The use of swimming paddles at maximal efforts would suppress the expected lactate production, which is a goal for certain training sets. At submaximal efforts paddles appear to be useful when one needs to reduce the stroking frequency and increase the stroke length without shifts in energy demands.

## Figures and Tables

**Table 1:** Anthropometric Characteristics

	Age (years)	Body Height (m)	Body Mass (kg)	BMI
N= 8	22.63±0.92	1.77±0.09	71.06±11.32	22.69±2.81

**Table 2:** Performance and Stroking Characteristics with (S.P.) and without (N.S.) paddles at 100% and 85% of maximum speed

n=8	Time (s)	Number of Strokes (strokes/distance)	Gliding Length (m)	Stroke Length (m/stroke)
S.P.-100%	82.46±11.44	69.25±19.07	18.09±5.14	1.22±0.20
N.S.-100%	82.46±11.44	74.25±18.16^*^	23.88±6.10^*^	1.05±0.14^*^
S.P.-85%	94.83±13.16	62.25±20.42	23.53±7.10	1.30±0.24
N.S.-85%	94.83±13.16	69.00±21.93^**^	23.99±5.32	1.16±0.21^**^

* One asterisk denotes significant differences between S.P.-100% and N.S.-100% trials

** Two asterisks denote significant differences between S.P.-85% and N.S.-85% trials S.P.= Swimming Paddles; N.S.= No Paddles
